# In Situ Local Resistance Analysis of Mechanical Degradation in All‐Solid‐State Batteries

**DOI:** 10.1002/advs.76825

**Published:** 2026-07-28

**Authors:** Hirotada Gamo, Yasushi Maeda, Yuji Yamagishi, Tetsu Kiyobayashi, Naoya Ishida, Zyun Siroma, Kentaro Kuratani, Nobuhiko Takeichi, Hikaru Sano

**Affiliations:** ^1^ Research Institute of Electrochemical Energy Department of Energy and Environment National Institute of Advanced Industrial Science and Technology (AIST) Ikeda Osaka Japan

**Keywords:** all‐solid‐state batteries, in situ atomic force microscopy, local resistance analysis, mechanical degradation mechanism, scanning spreading resistance microscopy

## Abstract

All‐solid‐state lithium‐ion batteries (ASSLIBs) have attracted considerable attention as next‐generation energy storage devices owing to their safety and high‐energy density. The electronic transport properties of cathode composites employing inorganic solid electrolytes depend strongly on their microstructural characteristics. Although mechanical failure due to the loss of interparticle contact between cathode active materials directly affects the electron transport pathways, it has been largely overlooked as a critical degradation mechanism in ASSLIBs. In this study, an in situ local resistance analysis technique based on scanning spreading resistance microscopy was developed to visualize changes in the local electronic resistance distribution within ASSLIBs. In situ observations of cathode composites during charging revealed electrical isolation of some cathode active materials during the initial stages of charging, followed by an increase in interparticle contact resistance at higher charging potentials. Furthermore, electrochemical simulations based on a three‐dimensional model qualitatively described the evolution of the experimental voltage profiles, highlighting mechanical degradation due to the interparticle contact loss between cathode active materials. The method proposed herein provides novel insights into mechanically induced electronic contact loss in active materials that cannot be evaluated by conventional topographical and morphological analyses.

## Graphical abstract

In this study, we developed an in situ scanning spreading resistance microscopy technique for all‐solid‐state batteries using sulfide solid electrolytes to visualize the evolution of local electronic resistance in NCM‐based cathode composites during charging. The observation revealed that some NCM particles become electrically isolated at early charging stages, followed by a pronounced increase in interparticle contact resistance at higher potentials, even in the absence of evident morphological changes.

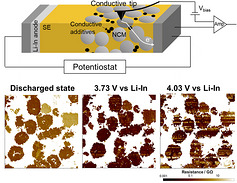

## 1 Introduction

All‐solid‐state lithium‐ion batteries (ASSLIBs) with inorganic solid electrolytes (SEs) are attracting attention as next‐generation batteries owing to their safety and high‐energy density [1]. Sulfide SEs, such as Li_6_PS_5_Cl (LPSCl) [2, 3] and Li_10_GeP_2_S_12_ (LGPS) [4], meet the requirements of high ionic conductivity over 10^−3^ S cm^−1^ at room temperature and high ductility. However, ASSLIBs with sulfide SEs suffer from cycle fading due to chemical and mechanical degradation [5]. Sulfide SEs exhibit narrow thermodynamic stability and hence are prone to reaction with cathode active materials (CAMs) [6, 7, 8]. This instability is widely considered the main cause of battery failure, and accordingly, the interfacial degradation between SEs and CAMs has been extensively investigated. Electrochemical impedance spectroscopy (EIS) and X‐ray photoelectron spectroscopy studies have revealed severe degradation at the LGPS/LiCoO_2_ interface [9, 10]. A recent study demonstrated that charging beyond 4.2 V vs. Li/Li^+^ facilitates the redox reaction between the delithiated LiNi_0.5_Co_0.2_Mn_0.3_O_2_ (NCM) and LPSCl, forming Li_2_S*_n_* and P_2_S*_x_* species [11]. The formation of decomposition products increases the overall impedance of the cell, and the decomposition can be partially mitigated via surface modification of the CAMs [12, 13]. In addition to the side reactions at the interface, space‐charge layer effects have been widely discussed as a possible contributor to interfacial resistance in ASSLIBs [14, 15]. Mechanical degradation within cathode composites has not been investigated as extensively as chemical degradation. Ceder et al. reported that voids and cracks formed near the CAMs after cycling result in contact loss at the CAM/SE interface [16]. Moreover, continuous cycling causes cumulative contact loss, leading to rapid capacity fading [17]. Although the contact area between CAMs is considerably smaller than that between CAMs and SEs, limited attention has been paid to the degradation of electronic transport arising from contact loss between CAMs. Several experimental and computational studies have highlighted the importance of connectivity between CAMs for battery performance in ASSLIBs [18, 19, 20, 21]. The connectivity of CAMs is related to the electrochemically accessible volume fraction of CAMs. Three‐dimensional (3D) simulations have revealed that electrodes with limited contact area between CAMs exhibit inhomogeneous current density distribution during operation and lower the capacity [21]. Although an EIS analysis based on the transmission line model implied the possibility of electronic contact resistance between NCM particles in cathode composites, its effects on battery performance remain unclear [22].

Scanning spreading resistance microscopy (SSRM), a type of conductive atomic force microscopy (AFM), is a powerful technique for acquiring microscopic electrical information at the sample surface [23, 24, 25, 26]. Otoyama et al. reported in an ex situ SSRM study that some NCM particles in the cathode composites lose contact with neighboring NCM particles after charging, leading to an increase in interparticle electronic resistance [27]. Moreover, an SSRM study by Kim's group demonstrated that a high‐pressure densification process enhances the electronic conduction paths via CAMs within electrodes [28]. Such local resistance analysis can help reveal electronic conduction mechanisms of electrodes used in ASSLIBs. In previous studies, we demonstrated that, when applied to composite materials, the local resistance measured by SSRM reflects not only the intrinsic resistivity of the particle directly beneath the probe but also the electronic contact resistance between that particle and its surroundings [29, 30]. In addition, an ex situ SSRM study of ASSLIBs with NCM cathode composites revealed that, when floated at 4.25 V vs. Li/Li^+^, the cell underwent no increase in local resistance due to mechanical degradation; in contrast, some NCM particles were electrically isolated when the cell was floated at 4.55 V [25]. Thus, mechanical degradation, such as interparticle contact loss and electrical isolation, can be visualized via SSRM. However, these ex situ characterization techniques may suffer from an equivocal interpretation of the change in the local resistance at the electrodes owing to discrepancies in securing an identical viewing field and voltage relaxation during sample transfer [31].

In this study, we develop in situ SSRM techniques for ASSLIBs and investigate the change in the local resistance of NCM cathode composites. The in situ SSRM analysis captures the increase in the contact resistance between NCM particles upon an increase in the charging potential. This reveals mechanical degradation through interparticle contact loss, which cannot be detected via scanning electron microscopy (SEM). Moreover, we compare the evolution of experimental differential capacity curves with 3D simulations to identify the effects of the evolution of local resistance on the battery performance. Our results indicate that mechanical degradation associated with the loss of interparticle electronic contact is a major factor in cycle fading over a broad operating voltage range.

## 2 Results and Discussion

### 2.1 In Situ Local Resistance Analysis

**FIGURE 1:**
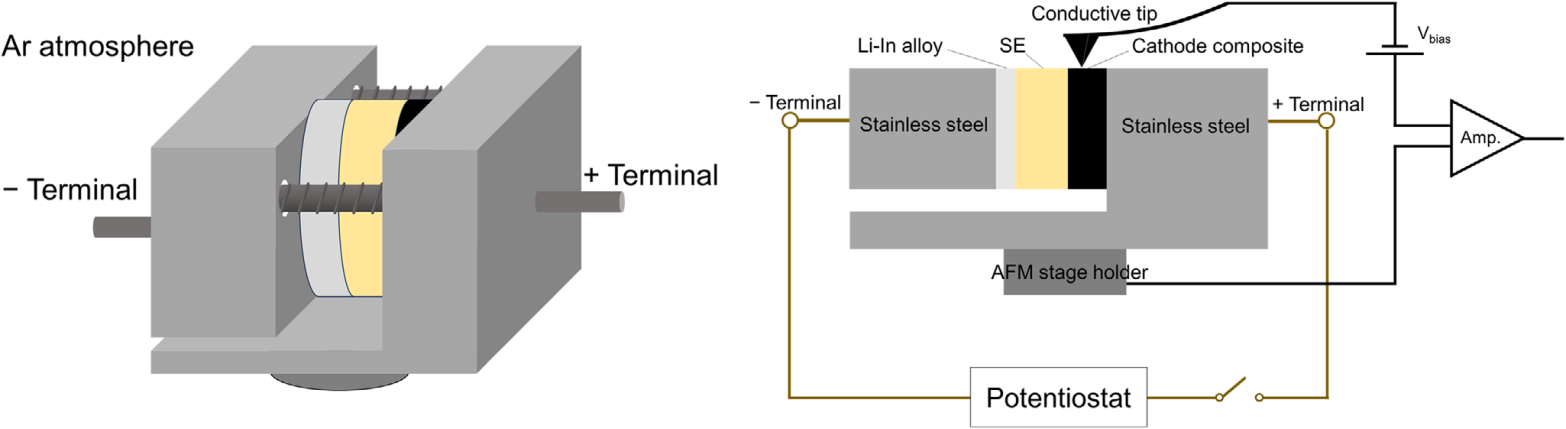
Schematic of the specially designed cell for in situ SSRM and the setup.

**FIGURE 2:**
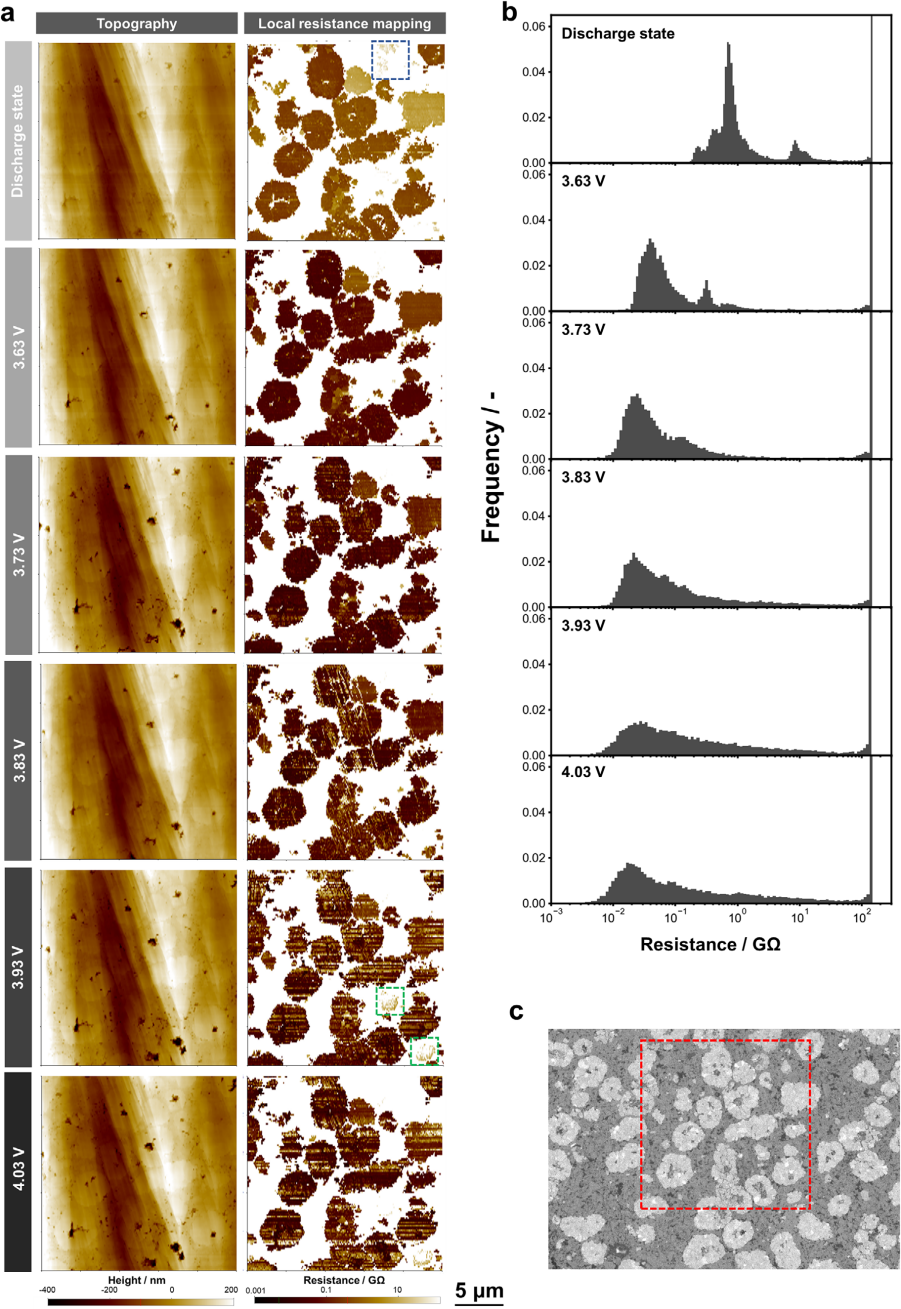
(a) Topographies and local resistance mapping images of cathode composites in the cells charged to different potentials. (b) Resistance histograms extracted from the local resistance mapping images shown in (a). (c) Cross‐sectional SEM image of the cathode composites charged to 4.03 V vs. Li‐In. The red dashed box indicates the SSRM observation region.

In situ AFM technique has been used to investigate the reaction mechanism and degradation behavior of ASSLIBs [31, 32, 33, 34, 35], but in situ SSRM studies investigating ASSLIBs with sulfide SEs are scarce. A challenge in this context is the construction of an observation environment that isolates the air‐sensitive sulfide SEs from moisture. Moreover, an appropriate confining stress must be applied during electrochemical operations to the cell used during the in situ observations. Therefore, we designed an attachment that can restrain the sample; a corresponding schematic is shown in Figure 1. The in situ observations were conducted using the SSRM instrument placed in a dried argon atmosphere. A previous SSRM study demonstrated that the effect of cross section processing on the local resistance of NCM particles in cathode composites is sufficiently small, because the local resistance of NCM with a well‐connected electronic conduction network is directly related to its intrinsic electronic resistivity even after cross section processing. Furthermore, damage to the cell used in the in situ SSRM observations due to sample preparation (e.g., ion milling for cross‐section processing and sample transfer) was negligible because the accessible capacity during in situ SSRM measurements reached 77% (213 mAh g^−1^) of the theoretical capacity at 4.03 V vs. Li‐In (corresponding to 4.65 vs. Li/Li^+^), as shown in Figure S1. Figure 2a shows the topography and local resistance mapping images of the cathode in the discharged state and charged states at 3.63, 3.73, 3.83, 3.93, and 4.03 V vs. Li‐In. The sizes of the voids observed inside the NCM secondary particles increased at higher charging potentials. A previous XRD study reported that the volume shrinkage of NCM from the discharged state to 3.63 V was 3.5vol% and that from 3.63 to 3.93 V was 3.2vol% [25]. Assuming spherical NCM particles with a diameter of 5 µm and isotropic volumetric contraction, these volume changes correspond to a reduction in particle diameter of approximately 50 nm. No obvious cracks within the electrodes were detected even after the cell was charged to 4.03 V, owing to the limited spatial resolution (1 pixel = 78 nm). The local resistance mappings indicated low resistance (brown color) in the NCM and AB regions and high resistance (bright color) in the SE regions. For the cathode composite in the discharged state, different resistance values were observed for each NCM particle. SSRM simulation and experimental studies have demonstrated that the local resistance in the CAM regions varies with the electronic conduction paths via conductive additives and with contact between CAM particles, even when the intrinsic electrical resistivities of the CAMs are identical [21, 29, 30]. The difference in the resistances of NCM observed in this study is attributed primarily to the variation in the contact resistance between the NCM particles. The overall resistance of the NCM regions decreased upon charging to 3.63 V. This decrease in the resistance suggests that the intrinsic electronic conductivity of NCM increases via a semiconductor‐to‐metal transition [36, 37]. In contrast, the local resistance of the NCM region in the upper right part (marked by a blue dashed box) of the observation area increased to an extremely high value (bright color) after charging from the discharged state to 3.63 V. This increase in the resistance suggests electronic isolation of NCM particles due to volume shrinkage. A previous ex situ SSRM study revealed that NCM particles are electrically isolated from the current collector after floating at 3.93 V [25], whereas the in situ SSRM results indicate the presence of isolated NCM particles, even during the initial charging stage. The overall resistance increased with an increase in the charging potential above 3.83 V. This increase is attributed to an increase in the contact resistance due to the volume shrinkage of the NCM particles. A previous in situ XRD study showed that the lattice parameter along the crystallographic *c*‐axis of NCM gradually expands up to ∼60mol% delithiation, beyond which the *c*‐axis abruptly contracts [38]. This lattice change above ∼60mol% delithiation leads to a drastic change in lattice distortion (the crystallographic lattice constant *c*/*a* ratio) [25]. As shown in Figure S1, 60mol% delithiation corresponds to a charged state between 3.73 and 3.83 V. At ∼60% delithiation (3.83 V), NCM undergoes an H2→H3 phase transition accompanied by abrupt volume shrinkage and changes in particle geometry, which can reduce the effective contact area between neighboring NCM particles and thereby increase the interparticle contact resistance. In contrast, the electrically isolated NCM in the bottom right region (marked by green dashed boxes) of the observation area reconnected electrically to the current collector after charging to 3.93 V. This reconnection may be attributed to the restored connectivity of NCM particles through the reversal of the direction of lattice distortion at high charging potentials. The reproducibility of the electronic isolation of NCM particles and their reconnection at higher potential was confirmed via a similar measurement using another cell (Figure S2). Figure 2b shows the resistance histogram extracted from the local resistance mapping images. The resistance distribution for the cathode composites charged up to 3.73 V shifted toward lower resistance with an increase in the charging potential. With an increase in the charging potential to 3.83 V or higher, the primary resistance distribution gradually dispersed toward the high‐resistance side. This change in resistance was also confirmed via a high‐resolution SSRM image (Figure S3), suggesting gradual electrical isolation of the NCM particles upon charging to high potentials. As shown in the topography and cross‐sectional SEM images, NCM particles are connected to each other through localized contact regions. Accordingly, minor structural changes can alter interparticle contact states, leading to electrical isolation and reconnection. Strauss et al. reported that, in conventional lithium‐ion batteries, the particle sizes of CAMs have a minimal influence on the effective electronic conductivity of electrodes, whereas in ASSLIBs, such microstructural characteristics have a significant influence on electronic transport [18]. Additionally, the fraction of CAMs in ASSLIBs influences an accessible capacity [19]. Thus, the electronic transport properties within the electrodes of ASSLIBs are highly sensitive to variations in their microstructure. The observed evolution of the local resistance highlights a mechanical degradation mechanism wherein microstructural changes accumulated during charging disrupt interparticle electronic contacts. Figure 2c shows a cross‐sectional SEM image of the cathode composites in the cell after in situ SSRM measurement at 4.03 V. The region marked by a red dashed box in the SEM image corresponds to the SSRM observation area. The black regions in the SEM image were predominantly occupied by AB, where the local resistance was close to that in the NCM regions. A previous SSRM study on ASSLIBs demonstrated that AB regions exhibit local resistance comparable to that of NCM, indicating discontinuous conductive paths of AB particles [29]. Thus, the local resistance observed in this work indicates the absence of AB percolation paths. In contrast, even trace amounts of AB particles are likely to reduce the electronic contact resistance between NCM particles and suppress their electronic isolation during charging [25, 29]. Some AB particles in the SEM image exhibited high resistance (bright color) in the local resistance mapping image, suggesting that they do not function as a conductive additive. This result indicates the potential for further improvement in battery performance through electrode microstructure design. Small NCM particles observed in the SEM image were electrically isolated, as shown in the SSRM results. This result implies that CAM powders finely pulverized beyond necessity easily disconnect from their electronic conduction pathways. Similar to the topography images, no obvious cracks at the NCM/SE interface and within secondary particles were observed in the SEM image. The absence of visible cracks in morphological characterization does not necessarily preclude mechanical degradation due to structural changes, such as lattice distortion and volme shurinkage. Local resistance analysis based on the SSRM technique allows semi‐quantitative evaluation of mechanical degradation that cannot be evaluated via morphological analysis.

### 2.2 Electrochemical Performance

**FIGURE 3:**
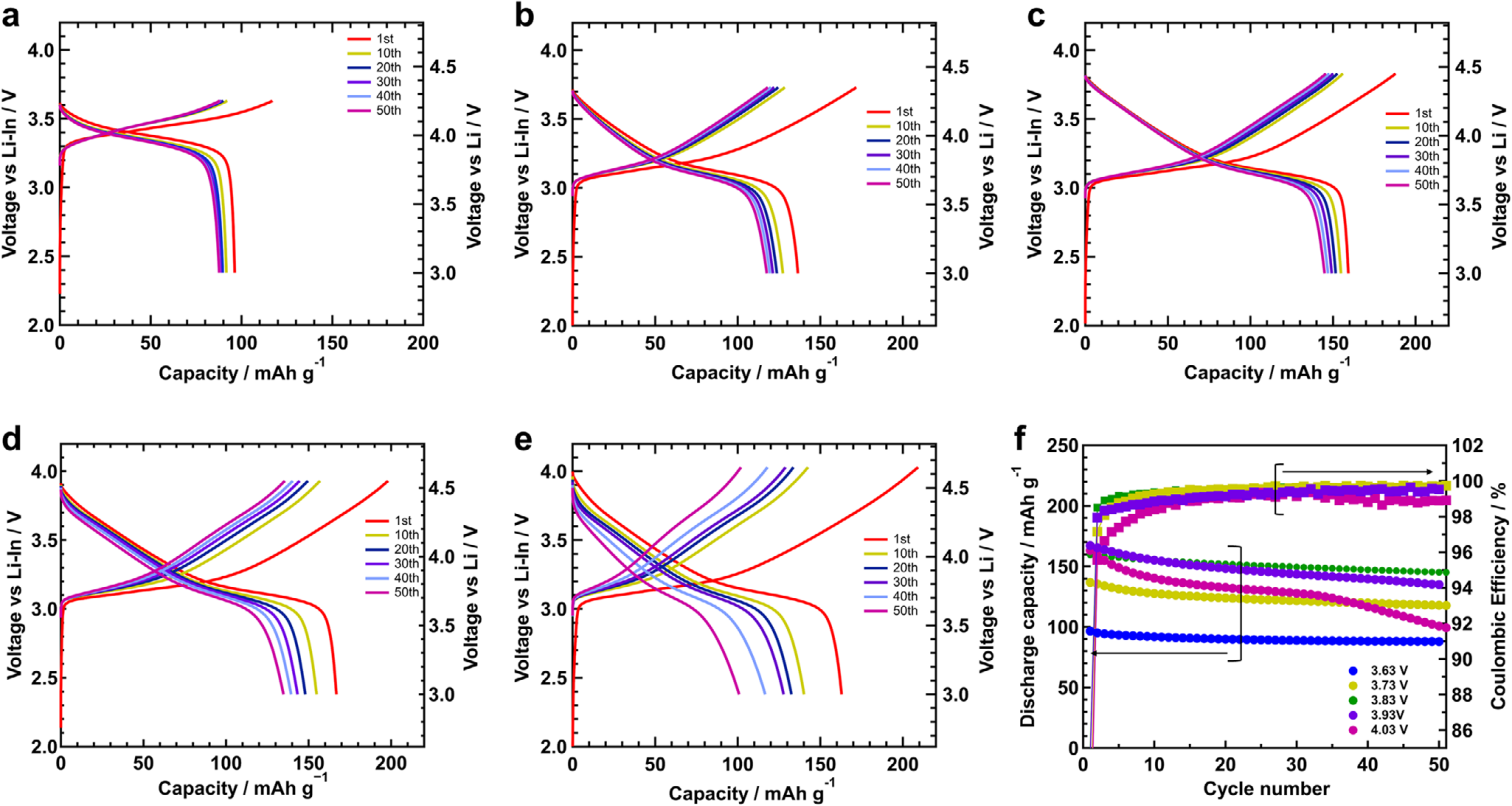
Galvanostatic voltage profiles of Li‐In|SE|NCM‐SE‐AB cells at upper cut‐off voltages of (a) 3.63 V, (b) 3.73 V, (c) 3.83 V, (d) 3.93 V, and (e) 4.03 V. (f) Cycle performance of Li‐In|SE|NCM‐SE‐AB cells.

**FIGURE 4:**
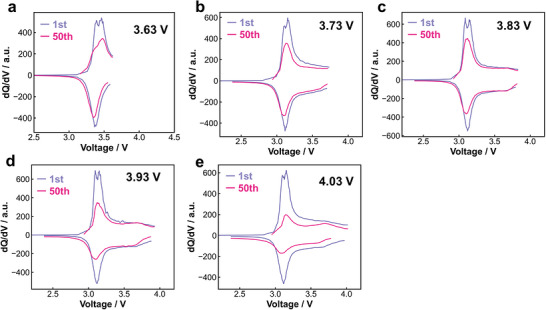
The *dQ*/*dV* curves of Li‐In|SE|NCM‐SE‐AB cells at upper cut‐off voltages of (a) 3.63 V, (b) 3.73 V, (c) 3.83 V, (d) 3.93 V, and (e) 4.03 V.

The effect of electronic contact resistance on battery performance was investigated via galvanostatic cycling at different upper cut‐off potentials (Figure 3a–e). The first charge capacities of cells operated at upper cut‐off potentials of 3.63, 3.73, 3.83, 3.93, and 4.03 V were 117, 172, 188, 198, and 209 mAh g^−1^, respectively. The discharge capacities of the five cells after discharging to 2.38 V were 97, 136, 159, 167, and 163 mAh g^−1^, corresponding coulombic efficiencies of 83%, 79%, 85%, 89%, and 78%, respectively. The cycle stabilities during 50 cycles are displayed in Figure 3f. The discharge capacities of the five cells at the 50th cycle were 88, 118, 145, 135, and 99 mAh g^−1^, respectively. The capacity loss during 50 cycles increased with an increase in the applied upper cut‐off potential (Figure S4). The electrode fabricated in this study exhibited an effective ionic conductivity of 1.6 × 10^−4^ S cm^−1^ and an effective electronic conductivity of 6.0 × 10^−6^ S cm^−1^ (Figure S5). To investigate the changes in resistance as a function of the charging potential, we measured the impedance spectra of the cells charged to each potential and analyzed the Nyquist plots and differentiation‐based Bode plots [39] (Figure S6). The semicircle with a peak frequency of 63 kHz enlarged with an increase in the charging potential, accompanied by a marginal decrease in the peak frequency. This increase can be attributed to the increase in the charge–transfer resistance at the NCM/SE interface. Moreover, the real part of the impedance in the high‐frequency region increased with an increase in the charging potential, which can be associated with electronic contact resistance between the NCM particles [22]. Although the EIS results highlight the chemical and mechanical degradation within electrodes to some extent, they offer a limited understanding of the effects of these degradations on battery performance. The chemical and mechanical degradation were further investigated by comparing the differential capacity (*dQ*/*dV*) curves at the 50th cycle with those at the first cycle (Figure 4). Peaks in the *dQ*/*dV* curves, caused by redox plateaus in the voltage profiles, correspond to phase transitions in the active materials owing to electrochemical intercalation. The degradation mechanisms, such as the loss of active materials and an increase in ohmic resistance, can be assessed by monitoring the location, magnitude, and width of these peaks. The peak at approximately 3.1 V during the first cycle was attributed to the H1→M phase transition (hexagonal to monoclinic) [40, 41, 42]. The shape of the main discharge peak in the 50th cycle was similar among the cells operated at 3.83 V or below and indicated marginal reductions in the magnitude and potential without changes in width compared to that at the first cycle. This similarity suggests that the degradation mechanism in the cells operated at 3.83 V or below is the same. At potentials higher than 3.83 V, the magnitude and potential at the discharge peak decreased further, with an increase in width.

### 2.3 Voltage Profile Simulation

**FIGURE 5:**
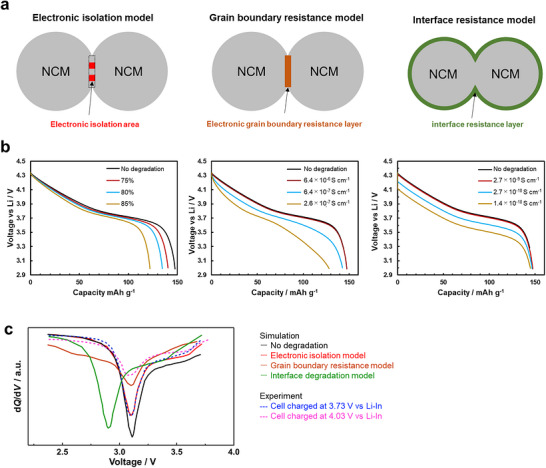
(a) Schematics of three degradation models for voltage profile simulation: electronic isolation model, grain‐boundary resistance model, and interface resistance model. (b) Corresponding simulated voltage profiles. (c) *dQ*/*dV* curves calculated from the voltage profiles. The experimental *dQ*/*dV* curves of the cells after 50 cycles in the voltage ranges from 2.38 to 3.73 V and to 4.03 V vs. Li‐In are shown for comparison.

We constructed three representative 3D electrodes with characteristic degradation modes and performed voltage profile simulations to elucidate the implications of the evolution observed in the *dQ*/*dV* curves. Figure 5a shows schematics of the three structural models: the electronic isolation model, the grain‐boundary resistance model, and the interface resistance model. The electronic isolation model includes an insulating layer between neighboring NCM particles at a given ratio to represent mechanical degradation due to the loss of electronic percolation among particles. The grain‐boundary resistance model incorporates a grain‐boundary resistance layer (1‐pixel thickness) with a given conductivity to represent mechanical degradation due to weakened contacts between NCM particles. The conductivity of the grain‐boundary resistance layer was set within a range of three orders of magnitude (6.4 × 10^−4^–2.6 × 10^−7^ S cm^−1^), corresponding to the three‐order‐of‐magnitude variation observed in the local resistance distribution of NCM particles. The interface resistance model introduces an interface resistance layer (1‐pixel thickness) with a given conductivity at the NCM/SE interface to account for chemical degradation resulting from interfacial side reactions between NCM and SEs. The simulated discharge voltage profiles for these models are shown in Figure 5b, and they qualitatively describe the influence of mechanical and chemical degradation on discharge performance. In the electronic isolation model, an increase in the ratio of insulation domains resulted in a decrease in discharge capacity, reflecting the loss of electrochemically accessible NCM particles. In the grain‐boundary resistance model, the overpotential increased gradually with the state of charge, reflecting pronounced Li‐concentration gradients within the electrodes, i.e., concentration overpotential. A previous simulation study has shown that electrodes with high electronic contact resistance between CAMs exhibit a significant capacity reduction due to severe concentration overpotential, resulting in inhomogeneous Li distribution along the electrode thickness direction [21]. This overpotential is mitigated by increasing the contact area between CAMs. In contrast, a constant overpotential over the entire SOC range without capacity loss was observed in the interface resistance model. To compare the experimental and simulated results, we selected three models that exhibited significant differences in their voltage profiles and calculated the *dQ*/*dV* curves from the simulated discharge voltage profiles. These models included the electronic isolation model with an 85% insulating fraction, the grain‐boundary resistance model incorporating a grain‐boundary resistance layer with a conductivity of 2.6 × 10^−7^ S cm^−1^, and the interface resistance model incorporating a layer with a conductivity of 1.4 × 10^−10^ S cm^−1^ at the NCM/SE interface (Figure 5c). The interface resistance model yielded a discharge peak that distinctly shifted to lower potentials relative to the cell without degradation. The electronic isolation model exhibited a discharge peak with a slightly lower magnitude and potential compared to the cell without degradation. The grain‐boundary resistance model resulted in a broader and lower‐magnitude peak compared to that in the electronic isolation model. The evolution of the experimental *dQ*/*dV* curves for the cells cycled below 3.83 V closely matched the *dQ*/*dV* curves predicted by the electronic isolation model. This result indicates that charging below 3.83 V leads to capacity loss due to electronic isolation of some NCM particles. This interpretation is supported by the electrically isolated NCM particles detected at 3.63 V in the SSRM observations. The evolution of the experimental *dQ*/*dV* curves for the cells cycled above 3.93 V showed an expansion of the peak width without a drastic peak shift, in good agreement with the grain‐boundary resistance model. This result demonstrates that the increased interparticle contact resistance at high potentials observed in SSRM is one of the key mechanical degradation mechanisms contributing to cycle fading. In addition, the overpotential (IR drop) observed in the cells cycled above 3.93 V suggests chemical degradation associated with increased NCM/SE interfacial resistance. These results indicate that mechanical degradation arising from the loss of electronic contact between NCM particles is a major contributor to capacity fading, whereas chemical degradation at the NCM/SE interface may provide an additional contribution, particularly under high‐voltage operation. Importantly, mechanical degradation related to electronic contact between NCM particles emerges as a key mechanism affecting the degradation of electrochemical performance across a wide operating voltage range. It should be noted that the dQ/dV analysis reflects the macroscopic electrochemical response of the entire cell, whereas SSRM provides microscopic resistance evolution. However, similar resistance evolution was consistently observed in multiple independently prepared cells, suggesting that the interparticle contact loss observed in this work occurs throughout the electrode with a certain statistical probability. As a result, the cumulative effect of such resistance evolution is expected to influence the overall electrochemical behavior of the cell, which is reflected in macroscopic measurements such as dQ/dV profiles and capacity. Previous voltage profile simulation studies using 3D models have typically assumed idealized electrode structures where active material particles are in perfect electronic contact, neglecting the electronic grain‐boundary resistance between CAMs [43, 44]. However, such a simplification does not capture certain features of experimental voltage profiles. In contrast, the 3D models considered in this study successfully reproduce the voltage profiles of cells operated at various upper cut‐off potentials. This model serves as a steady‐state degradation proxy and does not yet incorporate real‐time volumetric deformation. Recently, particle‐based electrochemical‐mechanical models, such as discrete‐element‐method simulations incorporating Li‐ion transport, electrochemical reactions, and charge‐induced expansion, have been reported for higly expanding Si‐based solid state battery anodes [45]. A dynamic structural‐evolution model incorporating real‐time deformation will be investigated in future work. These findings highlight the significant influence of electronic contact resistance between CAMs on battery performance and corroborate the in situ resistance analysis results. Thus, reliance on morphological analysis alone may obscure certain mechanical degradation mechanisms. Finding strategies to reduce and prevent the increase in the electronic contact resistance between active materials is critical for developing ASSLIBs with long lifetimes.

## 3 Conclusion

Herein, we developed an in situ SSRM‐based local resistance imaging technique for sulfide‐based ASSLIBs and investigated the mechanical degradation mechanism related to interparticle contact loss in NCM. The local resistance analysis directly visualizes mechanical degradation behavior: (i) electronic isolation of NCM particles during the early charging process (<3.83 V) and (ii) an overall increase in interparticle contact resistance at high potentials (>3.83 V). The electronic isolation of NCM particles is caused by subtle structural changes that cannot be detected from either surface morphology analysis and SEM observations. In response to the increase in resistance observed by the SSRM results, the cells operated at higher upper cut‐off potentials exhibited a decrease in capacity retention. Furthermore, the differential discharge capacity, *dQ*/*dV*, profiles of the cells charged to 3.83 V or higher differ from those of the cells charged to below 3.73 V, indicating changes in the degradation behavior. To unveil the origin of the evolution in the *dQ*/*dV* curves, we simulated voltage profile using three 3D models representing characteristic degradation modes. The voltage profile simulation of 3D model incorporating interparticle contact loss of NCM reproduced the experimental *dQ*/*dV* curves, providing further support for the degradation mechanism proposed from SSRM observations. Overall, this study revealed mechanical failure between NCM particles as a major contributor to capacity degradation over a wide operating voltage range in ASSLIBs, in addition to NCM/SE interface degradation that occurs under high‐voltage operation. These findings highlight the importance of understanding mechanical degradation behavior between CAMs for the development of long‐life ASSLIBs.

## Author Contributions

**H. G**.: conceptualization, data curation, formal analysis, investigation, visualization, methodology, and writing – original draft; **Y. M**.: conceptualization, data curation, formal analysis, investigation, visualization, methodology, funding acquisition, resource, supervision, writing – review & editing; **Y. Y**.: methodology, writing – review & editing; **T. K**.: conceptualization, project administration, methodology, supervision, and writing – review & editing; N. I.: investigation, methodology, and writing – review & editing; **Z. S**.: methodology, and writing – review & editing; **K. K**.: conceptualization, resource, methodology, and writing – review & editing; **N. T**.: supervision and writing – review & editing; **H. S**.: conceptualization, project administration, funding acquisition, resource, supervision, and writing – review & editing.

## Conflicts of Interest

The authors declare no conflicts of interest.

## Supporting information



The authors have cited additional references within the Supporting Information [21, 25, 30, 46–48].
**Supporting file**: advs76825‐sup‐0001‐SuppMat.pdf

## Data Availability

The data that support the findings of this study are available from the corresponding author upon reasonable request.
