# Supplementary material for: In Situ Local Resistance Analysis of Mechanical Degradation in All‐Solid‐State Batteries
Source: Adv Sci (Weinh). 2026 Jul 28;13(59):e76825. doi: 10.1002/advs.76825 (PMC13410802; doi:10.1002/advs.76825)
Supplement: Supplementary file 1 [file ADVS-13-e76825-s001.pdf]

Supporting Information

In Situ Local Resistance Analysis of Mechanical Degradation in All-Solid-State Batteries

Hirotada Gamo[1,*], Yasushi Maeda[1,*], Yuji Yamagishi[1], Tetsu Kiyobayashi[1], Naoya Ishida[1], Zyun Siroma[1], Kentaro Kuratani[1], Nobuhiko Takeichi[1], Hikaru Sano[1,*]

[1] Research Institute of Electrochemical Energy, Department of Energy and Environment, National Institute of Advanced Industrial Science and Technology (AIST), 1-8-31 Midorigaoka, Ikeda, Osaka 563-8577, Japan

*Corresponding authors: h.gamou@aist.go.jp, y-maeda@aist.go.jp, and hikaru.sano@aist.go.jp

Experimental Section

Fabrication of cathode composites

$LiNi_{0.5}Co_{0.2}Mn_{0.3}O_2$ (NCM)-solid electrolyte (SE)-acetylene black (AB) cathode composites were prepared by gently mixing with NCM (Sumitomo Metal Mining) with $LiNbO_3$-coating, argyrodite-structured $Li_{7-x}PS_{6-x}Cl_x$ (LPSCl, $x \sim 1$, D50 = 0.7 μm, Mitsui Kinzoku), and AB (Denka) at a weight ratio of 70:30:3 using a mortar for 10 min. The $LiNbO_3$ buffer layer was used to decrease the interfacial resistance at the NCM/SE interface.

Electrochemical characterization

Li-In|SE|NCM-SE-AB cells for the galvanostatic cycling tests were assembled as follows. SE powder (80 mg) was filled into the cylinder with two stainless-steel rods and pressed at a uniaxial pressure of 360 MPa to obtain pellets with diameters of 10 mm at room temperature. NCM-SE-AB cathode composite (20 mg) was spread over one side of the SE layer and pressed at a pressure of 360 MPa at room temperature. Indium and lithium foils were placed on the other side of the SE layer and pressed at 90 MPa at room temperature. The fabricated cells were allowed to rest for 5 h and then subjected to a one-cycle galvanostatic cycling as a pre-treatment at 0.064 mA cm^{-2} in the voltage range of 2.38–3.63 V versus Li-In at 25 °C using a charge–discharge device (TOSCAT-3100; Toyo System). For electrochemical impedance spectroscopy (EIS) measurements, the cells were first charged to 3.63, 3.73, 3.83, 3.93, and 4.03 V vs Li-In in the constant current-constant voltage mode and subsequently returned to 3.13 V. The impedance spectra were then recorded using an electrochemical workstation (VMP-300, Bio-Logic Science Instruments) at 3.13 V over a frequency range of 1 MHz to 10 mHz with an alternating voltage amplitude of 10 mV.

Material characterization

After the all-solid-state cells were preprocessed, flat cross-sections of the cathode composites were prepared by Ar-ion beam milling under cooling at −100 °C using an IB-19520CCP cross-section polisher (JEOL). The scanning spreading resistance microscopy (SSRM) measurements were conducted for the cross-sections of the cathode composites of cells charged to potentials of 3.63, 3.73, 3.83, 3.93, and 4.03 V in the constant current–constant voltage mode. SSRM was conducted under a sample bias voltage of 2.0 V and pin-point mode scanning control using atomic force microscopy equipment (NX10, Park Systems). A strong loading force of 600 nN was applied during SSRM measurements, which minimizes the contact resistance between the tip and the materials. The tip radius of the Pt-coated cantilever of SSRM (ElectriTap150-G, BudgetSensors) was 25 nm. The fabricated cell was constrained by the attachment for in situ SSRM under a pressure of approximately 20 MPa. During SSRM measurements, the cell is electrically isolated from the potentiostat, and the SSRM bias is applied independently through the atomic force microscopy-based measurement circuit. All treatments were performed under an Ar atmosphere (< 0.1 ppm O_2, 0.1 ppm H_2O). Cross-sectional images of the cathode composites were obtained using field emission scanning electron microscopy (Regulus 8220; Hitachi High-Tech) at 5 kV with a secondary electron image. After in situ SSRM observation, the sample was transferred from the glovebox into the instrument under an inert atmosphere using a transfer vessel.

Discharge profile simulation

The electrochemical discharge profiles were simulated using the BatteryDict module within GeoDict software. The 3D battery model for all-solid-state half-cells was based on the 3D structure of batteries comprising lithium metal (400 × 400 × 8 voxels), LPSCl SE layer (400 × 400 × 50 voxels), and NCM-LPSCl cathode composites (400 × 400 × 100 voxels) (see Fig. S7). The edge of one voxel corresponds to 0.2 µm. A virtual electrode was created by filling the spherical CAMs with a radius R of 2.5 µm and then filling the voids within the sphere-filled structures with polyhedral SEs of R = 1 µm. The volume ratio of NCM, LPSCl, and voids was set to 40:50:10. The contact area between NCM particles was 3.73 cm^2 (Fig. S7c). The bulk electronic conductivity of NCM was 6.4×10^{-4} S cm^{-1}, and the bulk ionic conductivity of LPSCl was 2.7×10^{-3} S cm^{-1}. Other parameters determining the transport phenomena are summarized in Supplementary Table S1. The simulation was initiated from the state in which all the active material particles were charged.

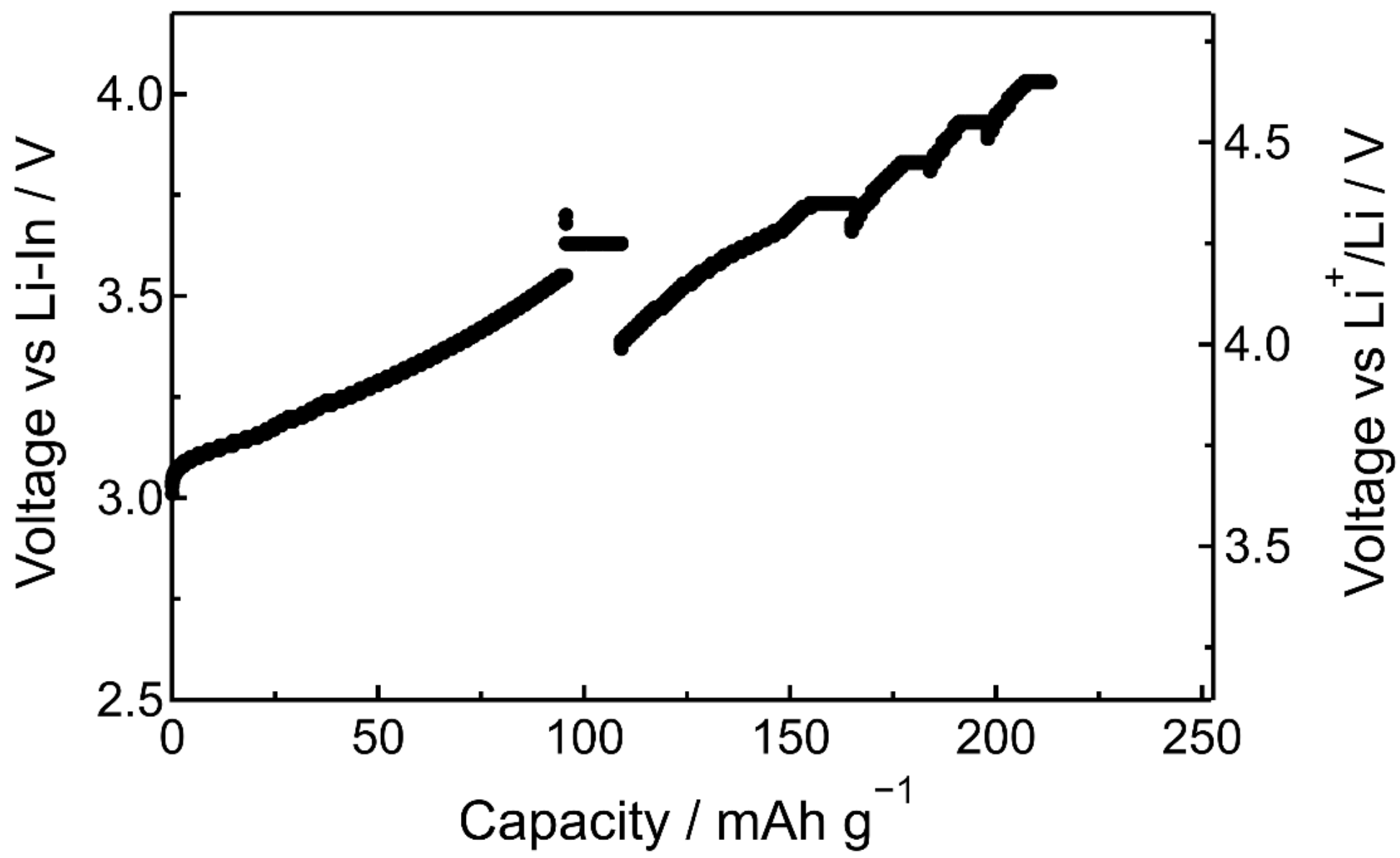

Figure S1. Voltage profiles during in situ SSRM measurement on the Li-In|SE|NCM-SE-AB cell.

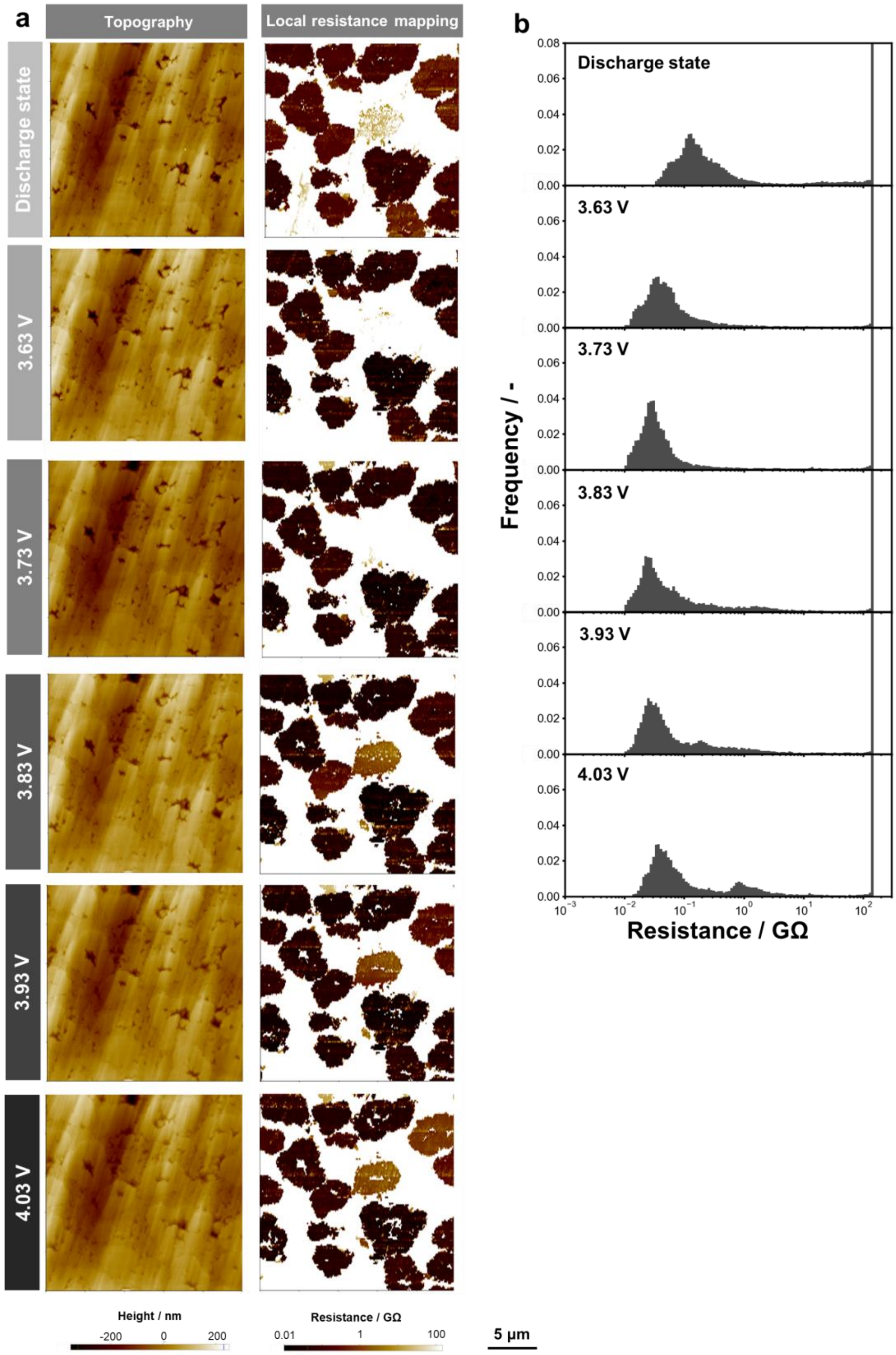

Figure S2. (a) Topographies and local resistance mapping images of the cathode composites of cells charged to different potentials. (b) Resistance histograms extracted from the local resistance mapping images shown in (a).

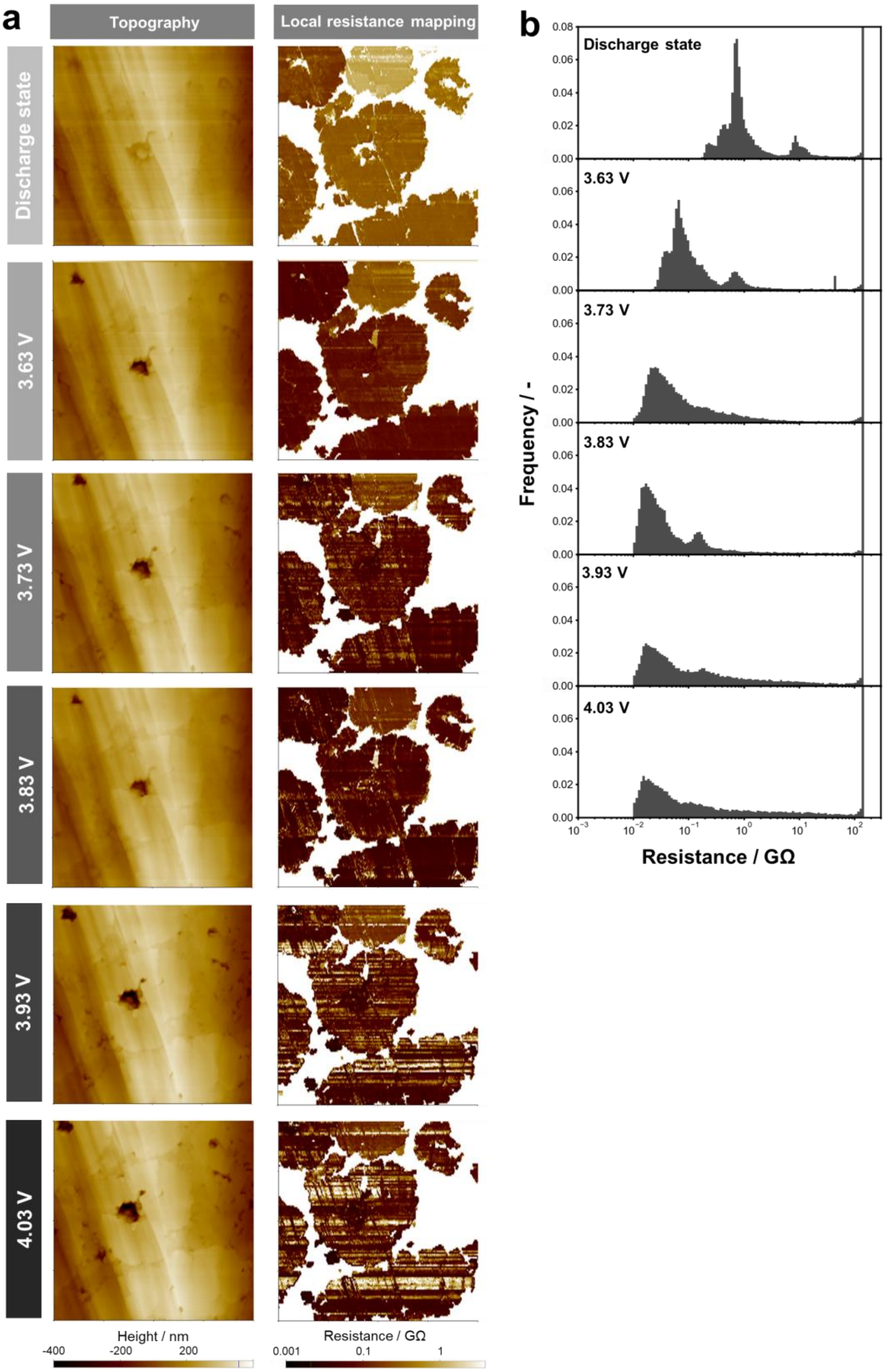

Figure S3. (a) High-resolution topographies and local resistance mapping images of the cathode composites of cells charged to different potentials. (b) Resistance histograms extracted from the local resistance mapping images shown in (a).

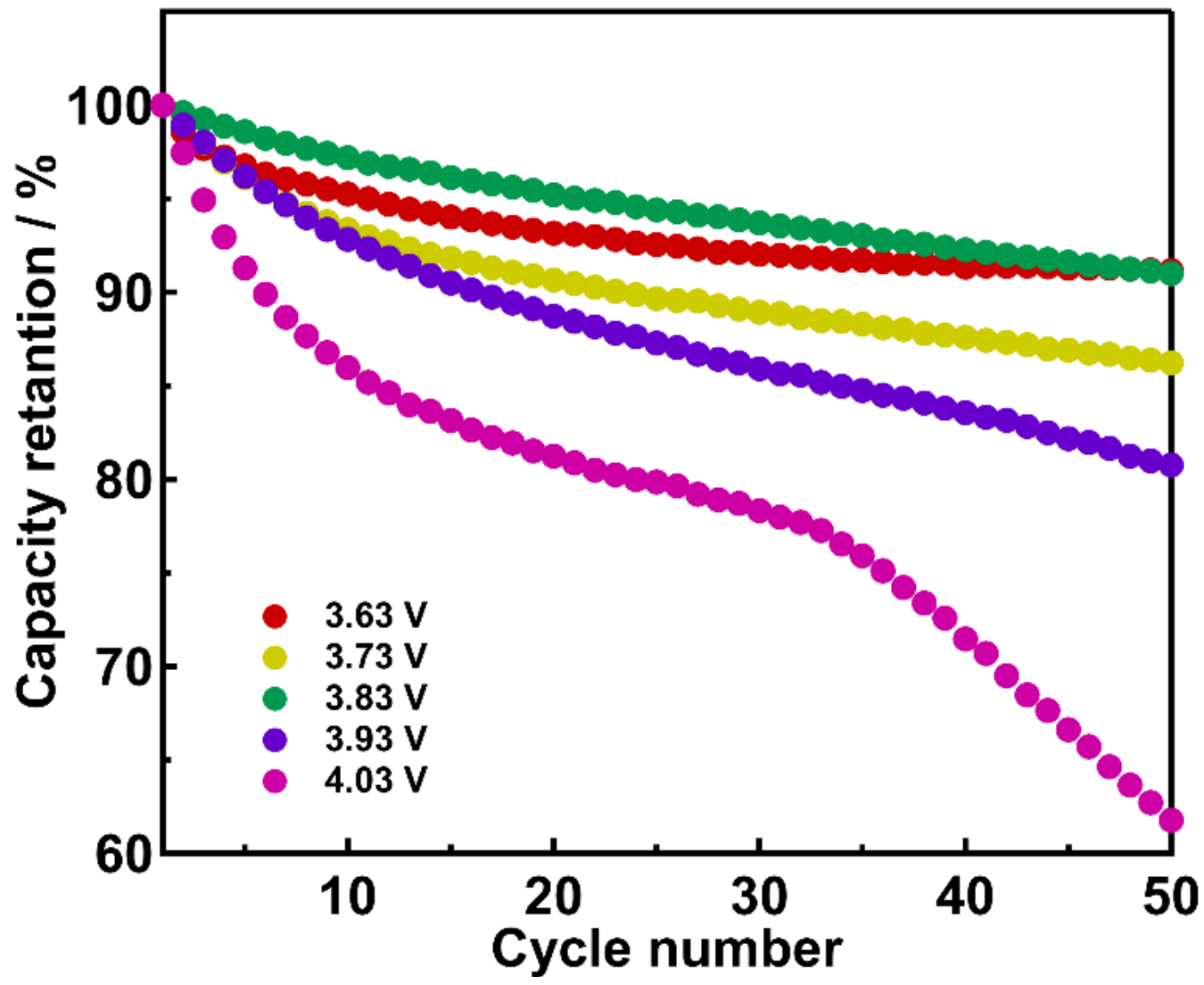

Figure S4. Capacity retention during 50 cycles for all-solid-state cells operated at various upper cut-off potentials.

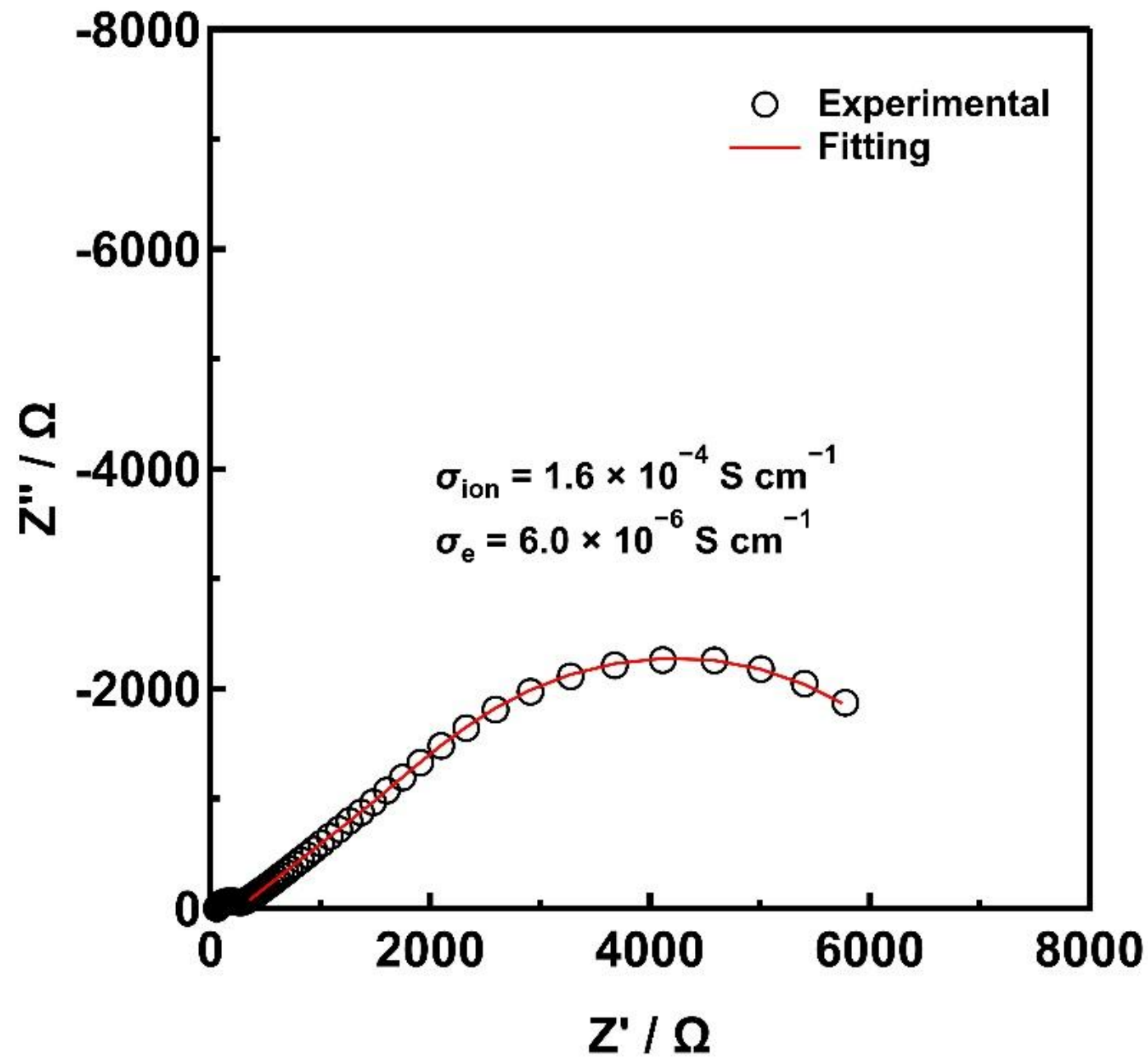

Figure S5. EIS results for the NCM cathode composites recorded at a voltage amplitude of 10 mV in the frequency range between 1 MHz and 10 mHz. The EIS results were fitted with a transmission line model based on electron–electron connection.

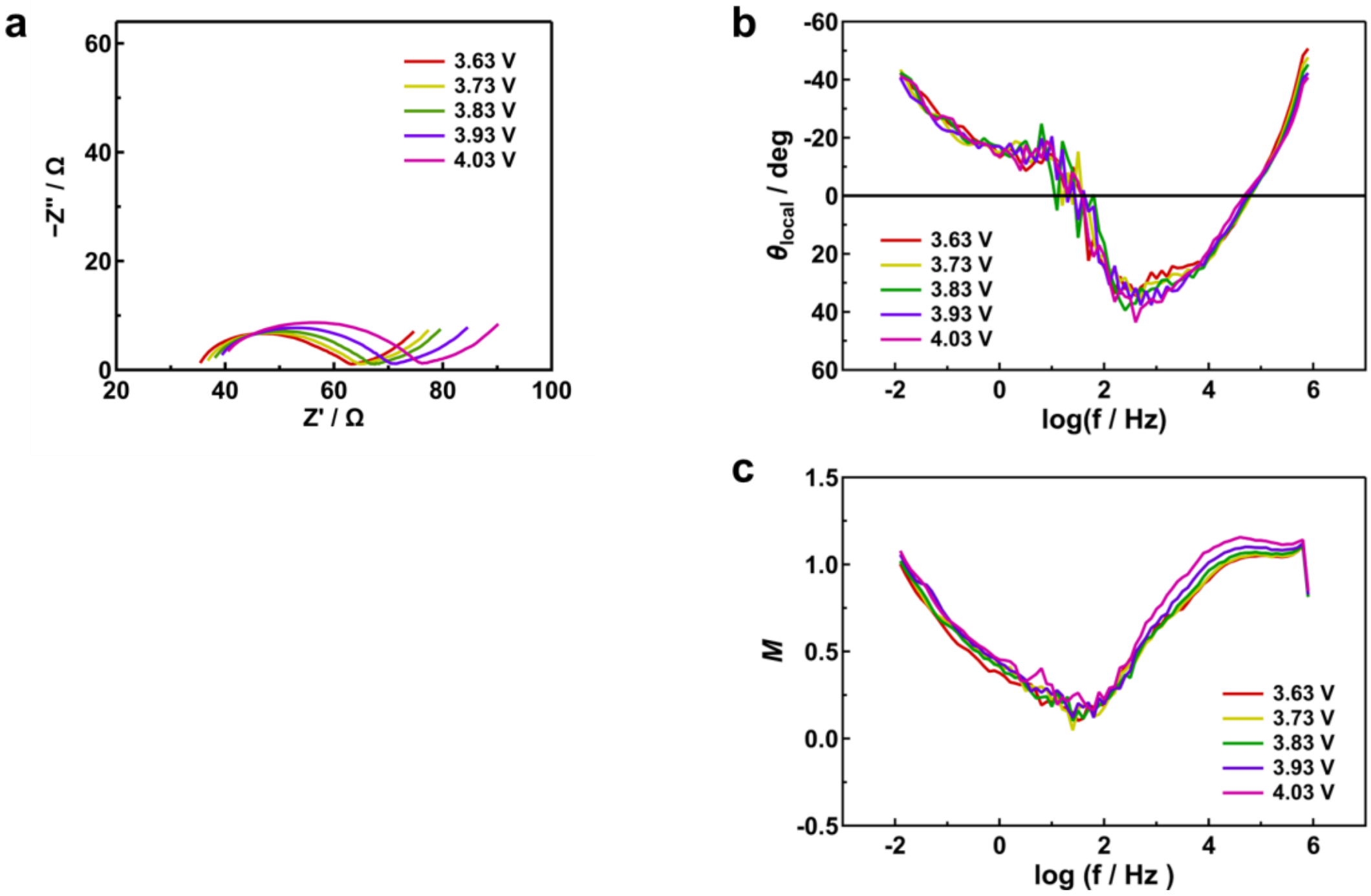

Figure S6. (a) Nyquist plots, (b) differential-based Bode plot, and (c) *M*-curves of cells charged to different potentials. The EIS spectra were recorded at a cell voltage of 3.13 V versus Li-In.

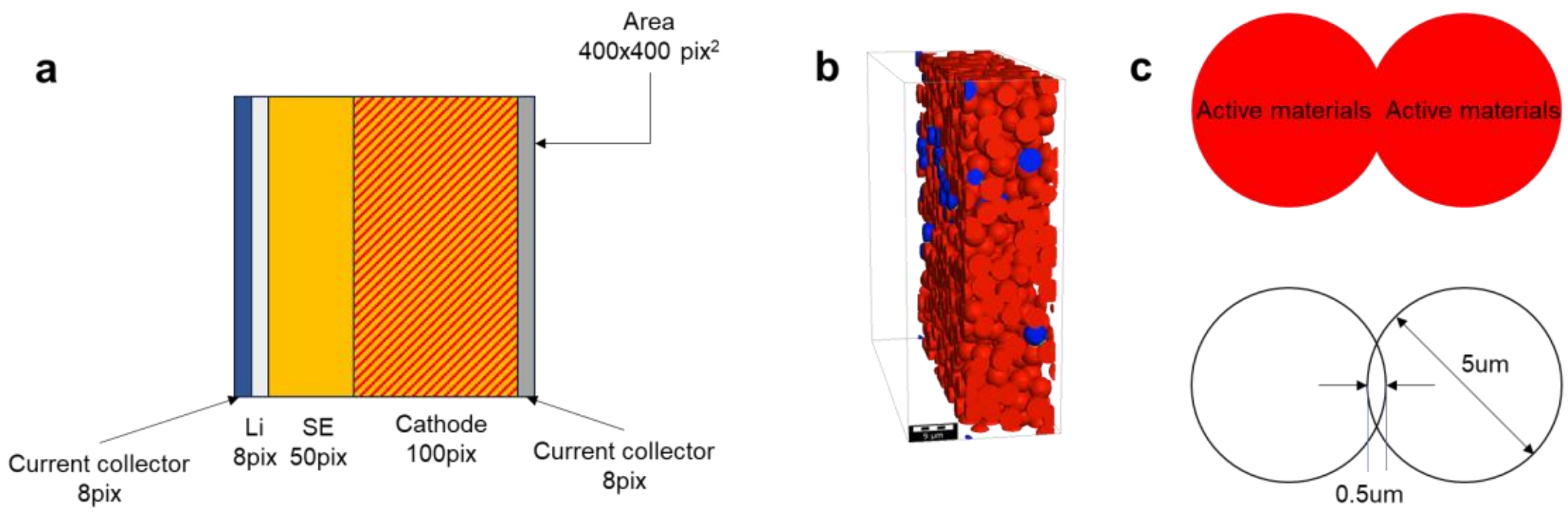

Figure S7. (a) Schematics of the 3D battery model for an all-solid-state half-cell. (b) The microstructure of the cathode composite with visualized cathode active material. The red and blue particles represent NCM particles connected to and disconnected from current collectors, respectively. (c) Contact state of active material particles (overlapping area).

Table S1. Parameters used in the discharge profile simulation.

Material	Parameter	Value
NCM	Electronic conductivity (σ) [30]	6.4×10^{-4} (S cm^{-1})
	Maximum lithium concentration [49]	31029 (mol m^{-3})
	Ionic diffusivity [46]	1.04×10^{-14} (m^2 s^{-1})
	Butler–Volmer rate constant [47]	5×10^{-7} (Am$^{2.5}$ mol$^{-1.5}$)
	Open circuit potential of the active material vs. lithium	[25]
LPSCl	Ionic conductivity (σ_{se}) [48]	2.7×10^{-3} (S cm^{-1})
	Ionic diffusivity [21]	3×10^{-9} (m^2 s^{-1})
	Lithium transference number	0.99
Lithium	Butler–Volmer rate constant [47]	20 (A mmol$^{-0.5}$)

Reference

[21] Y. Maeda, H. Gamo, Y. Yamagishi, H. Sano, Z. Siroma, N. Takeichi, T. Kiyobayashi, "Impact of Interparticle Contact on Discharge Capacity in All-Solid-State Batteries: A 3D Simulation Approach" Journal of Vacuum Science & Technology B 2025, 43, 052401. https://doi.org/10.1116/6.0004408.

[25] H. Gamo, Y. Maeda, K. Kuratani, Y. Yamagishi, T. Kiyobayashi, Z. Siroma, N. Takeichi, and H. Sano, "Degradation Processes in Positive Electrode Composites for All-Solid-State Lithium-Ion Batteries Visualized by Scanning Spreading Resistance Microscopy" Small methods, 2025, 9, 2500080–2500088. https://doi.org/10.1002/smtd.202500080

[30] H. Gamo, Y. Maeda, T. Kiyobayashi, Z. Siroma, and H. Sano, "Elucidating the mechanism of microscopic conduction in cathode composites for all-solid-state batteries through scanning spreading resistance microscopy" J. Mater. Chem. A, 2024, 12, 14380–14388. https://doi.org/10.1039/D4TA01634C

[46] H.-J. Noh, S. Youn, C. S. Yoon, and Y.-K. Sun, "Comparison of the structural and electrochemical properties of layered $Li[Ni_xCo_yMn_z]O_2$ (x = 1/3, 0.5, 0.6, 0.7, 0.8 and 0.85) cathode material for lithium-ion batteries" *Journal of Power Sources* 2013, *233*, 121–130, https://doi.org/10.1016/j.jpowsour.2013.01.063.

[47] X. Lu, X. Zhang, C. Tan, T. M. M. Heenan, M. Lagnoni, K. O'Regan, S. Daemi, A. Bertei, H. G. Jones, G. Hinds, J. Park, E. Kendrick, D. J. L. Brett, and P. R. Shearing, "Multi-length scale microstructural design of lithium-ion battery electrodes for improved discharge rate performance" *Energy & Environmental Science* 2021, *14*, 5929–5946.

https://doi.org/10.1039/D1EE01388B.
[48] A. Bielefeld, D. A. Weber, and J. Janek, "Modeling Effective Ionic Conductivity and Binder Influence in Composite Cathodes for All-Solid-State Batteries" *ACS Applied Materials & Interfaces* 2020, *12*, 12821–12833. https://doi.org/10.1021/acsami.9b22788.
[49] the Material Database in GeoDict
